# Validation of the Spanish Version of the Headache Impact Test (HIT‐6) in Patients With Episodic Migraine

**DOI:** 10.1002/brb3.70515

**Published:** 2025-05-05

**Authors:** Yaiza Colorado‐Martín, Daniel Pecos‐Martín, Nerea de Miguel‐Hernando, Rubén Cámara‐Calmaestra, Daniel Rodríguez‐Almagro, Alejandro Ferragut‐Garcías, Eduardo Castro‐Martín, Alexander Achalandabaso‐Ochoa

**Affiliations:** ^1^ Department of Nursing and Physiotherapy Universidad de Alcalá Alcalá de Henares Spain; ^2^ Department of Surgery, Ophthalmology, Otorhinolaryngology and Physical Therapy, Faculty of Health Sciences University of Valladolid Soria Spain; ^3^ Department of Health Sciences Universidad de Jaén Jaén Spain; ^4^ Department of Nursing, Physical Therapy and Medicine University of Almería Almería Spain; ^5^ Department of Nursing and Physiotherapy University of Islas Baleares Palma Spain; ^6^ Department of Physiotherapy, Faculty of Health Sciences University of Granada Granada Spain

**Keywords:** headache, impact evaluation, migraine disorders, questionnaires

## Abstract

**Introduction:**

The Headache Impact Test (HIT‐6) questionnaire is commonly utilized to assess the impact of headaches in both clinical settings and research. To date, no validated Spanish version of this tool has been published.

**Objective:**

This study seeks to evaluate the psychometric properties of the Spanish version of the HIT‐6 questionnaire for use in patients experiencing episodic migraine.

**Methods:**

We conducted a cross‐sectional study aimed at validating this measurement instrument. A total of 100 subjects, both male and female, aged 18 to 65 years, diagnosed with episodic migraine, were included in the analysis. Construct validity was assessed using principal component analysis, test‐retest reliability via the intraclass correlation coefficient (ICC), internal consistency, and convergent validity against the 12‐Item Short Form Health Survey and the Migraine Disability Assessment.

**Results:**

The principal component analysis revealed a two‐component structure. The overall HIT‐6 scale demonstrated strong test‐retest reliability ([ICC = 0.89; 95% CI = 0.83–0.92]), with high reliability for the indirect subscale [(ICC = 0.87; 95% CI = 0.81–0.91)] and excellent reliability for the direct subscale [(ICC = 0.90; 95% CI = 0.85–0.93)]. Internal consistency was also robust (Cronbach's *α* = 0.834), and the questionnaire showed a significant correlation with MIDAS (r = 0.512; *p* < 0.001), as well as a moderate correlation with the physical (r = ‐0.326; *p* < 0.05) and mental factors (r = ‐0.429; *p* < 0.001) of the SF‐12.

**Conclusions:**

The Spanish adaptation of the HIT‐6 questionnaire is a reliable and valid tool for evaluating the impact of episodic migraine on patients' quality of life, confirming the validity of both subscales.

## Graphical abstract

The Headache Impact Test (HIT‐6) questionnaire is a valid and reliable tool for assessing the impact of episodic migraine in the Spanish population. These findings may help enhance clinical evaluation and treatment strategies for episodic migraine in Spanish population.

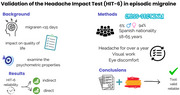

## 1 Introduction

According to the Global Burden of Disease Study 2021 (GBD), it is estimated that around 15.2% of the global population suffers from migraine (18.9% among women and 11.4% among men) (GBD 2021 Nervous System Disorders Collaborators 2024; Raggi et al. 2024), a condition recognized for its disabling nature (Dodick 2018). Globally, it ranks as the second leading cause for both sexes combined, representing 4.73% of health loss expressed in years lived with disability (Raggi et al. 2024), mainly in individuals aged 15 to 49 years (Waliszewska‐Prosół et al. 2024). Additionally, it stands out as the leading cause among the female population within this age range (Raggi et al. 2024).

In Spain, although epidemiological studies on this disorder are limited, it has been identified as a significant public health issue, affecting 11.02% of the population (Fernández‐de‐Las‐Peñas et al. 2010). Migraine is classified as a neurological disorder originating from the nervous system (Dodick 2018; Buse et al. 2019). However, it not only involves neurobiological symptoms but also has psychosocial, personal, and economic implications (Tana et al. 2024). It typically presents with unilateral, pulsatile pain in the frontotemporal region moderate to severe intensity and heightened sensitivity to movement as well as visual, auditory, and other sensory stimuli and is exacerbated by physical exercise (Goadsby et al. 2017; Olesen 2018; Tana et al. 2024).

Migraine is one of the most significant causes of disability among patients with primary headaches, leading to functional impairment that encompasses both physical and psychological aspects. Some individuals may experience an increase in the frequency of migraine attacks over time, which can lead to a progression toward episodic migraine (fewer than 15 attacks per month) or chronic migraine (at least 15 days of headache per month for more than three months, with at least eight days featuring migraine characteristics). Therefore, this increase in the frequency of attacks significantly impacts the patient's vitality, limiting their participation in recreational activities and social life (Pradeep et al. 2020).

Some researchers have determined that assessing the impact of migraine on patients extends beyond merely considering the frequency of episodes or the number of attack days (Yang et al. 2011; Rendas‐Baum et al. 2014). The clinical evaluation of how episodic migraines affect patients is crucial, both for informing treatment strategies in clinical practice and for advancing research on this disorder (Yang et al. 2011).

The Headache Impact Test (HIT‐6) serves as a valuable tool for assessing the impact of migraine, designed specifically to quantify the effects of headaches (Martin et al. 2004) and facilitate the detection and monitoring of migraine in individuals experiencing headache disorders, both in clinical and research contexts (Yang et al. 2011). Although widely used in Spanish‐speaking populations, a validated Spanish version of the HIT‐6 questionnaire is notably absent. The existing Spanish adaptation was validated using a sample of Mexican subjects (Martin et al. 2004), making it applicable for the Latin American demographic but inadequate for the Spanish population due to linguistic variations (the excessive use of “usted” in each question, the word “severe” is somewhat confusing, and the research team used “intense”). In light of the prevalent use of this assessment tool and the lack of a validated Spanish version tailored for Spain, our study aimed to investigate the psychometric properties of the HIT‐6 for use among Spanish patients with episodic migraine.

**Summary**

The HIT‐6 questionnaire is frequently used in the Spanish‐speaking population, but the version used is valid only for Latin American speakers, not for Spanish speakers. A validation study of the psychometric properties of the HIT‐6 questionnaire was conducted in a Spanish population with episodic migraine. It was concluded that the Spanish version of the HIT‐6 is a valid and reliable tool for assessing the impact of headache in the Spanish population with episodic migraine.

## 2 Material and Methods

### 2.1 Participants

The study involved volunteers aged 18 to 65 years who had been diagnosed with episodic migraine by a doctor/neurologist. All participants provided informed consent prior to enrollment. Before joining the study, each volunteer was evaluated by a physician to ensure they met the criteria established in the Third Edition of the International Classification of Headache Disorders (Headache Classification Committee of the International Headache Society 2018). The following exclusion criteria were applied: (a) currently undergoing preventive physiotherapy treatment, (b) use of prophylactic migraine medication, (c) pregnancy or breastfeeding, (d) presence of neurological, systemic, or mental disorder, (e) degenerative bone diseases or metabolic/musculoskeletal conditions that could pose a risk to the vertebral artery or cause vertigo, dizziness, or uncontrolled blood pressure, (f) use of specific medications, and (g) inadequate proficiency in Spanish. In the end, a total of 100 participants completed all study procedures.

**FIGURE 1:**
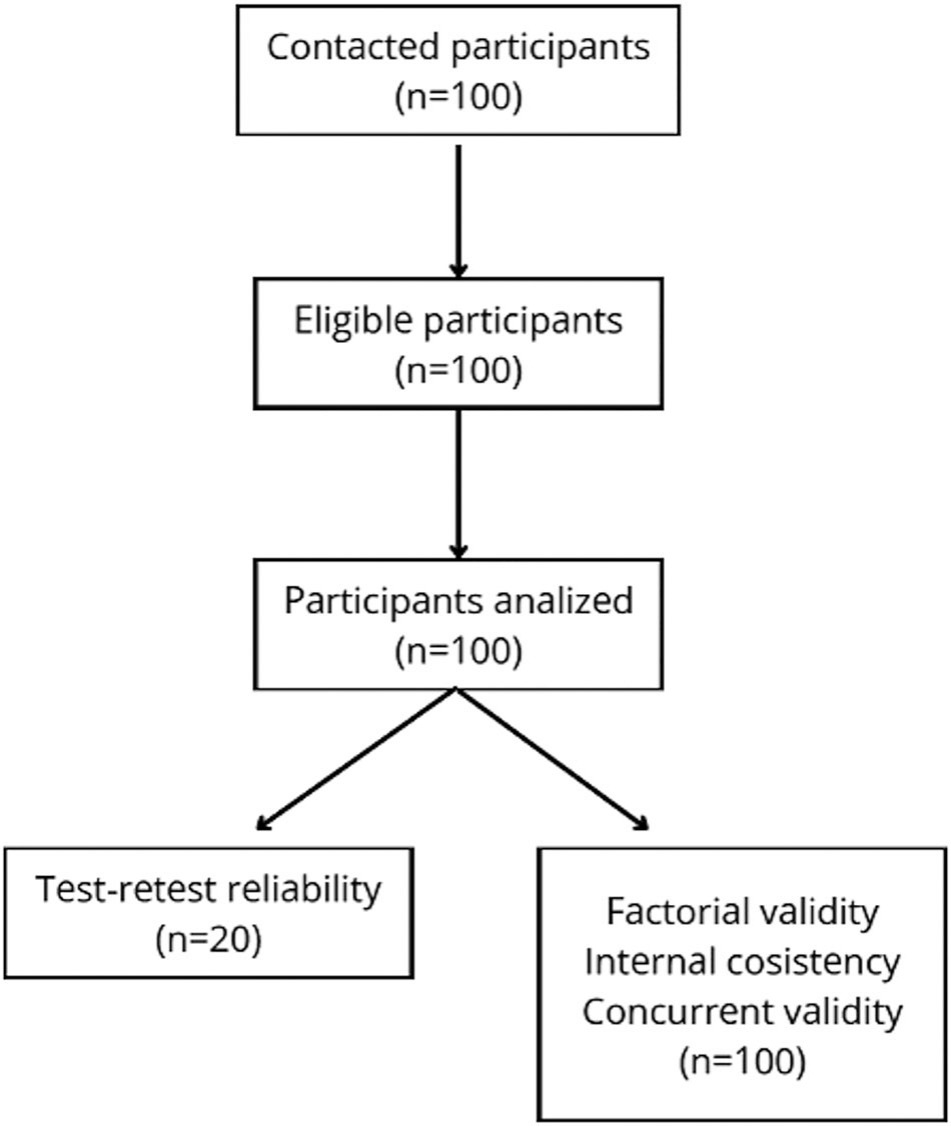
Flow diagram of the sample.

To calculate the sample size, we adhered to the recommendation of including at least five participants per questionnaire item, with an optimal target of ten participants per item and a minimum total of 100 participants to assess factor validity and internal consistency (Kline 2014). Furthermore, guidelines suggesting a minimum of 20 participants for reliability testing and at least 40 participants for the evaluation of convergent validity were followed (Hobart et al. 2012). The participant selection process is illustrated in Figure 1.

### 2.2 Procedure

This study received approval from the Ethics Committee of the University of Alcalá (reference number CEID/2022/47085) and was conducted in compliance with the Helsinki Declaration, good clinical practices, and all relevant laws and regulations. All participants received an information sheet about the study, which stated that they could withdraw from the study at any time without any penalty. They then provided their informed consent in writing and online prior to their involvement in the study.

Two bilingual experts independently translated the English version of the HIT‐6 questionnaire (Kosinski et al. 2003) into Spanish, adhering to the guidelines outlined by the International Quality of Life Assessment Project for cross‐cultural translations (Bullinger et al. 1998). Following this, a consensus was reached between the translators and the research team to produce a preliminary forward translation ensuring that the language used was similar, simple, and understandable that it followed a correct grammatical structure; and that the language level was comparable to Spanish. Subsequently, two bilingual experts carried out a back‐translation of the agreed‐upon Spanish version into English. This English translation was then compared to the original HIT‐6 questionnaire to ensure semantic, linguistic, conceptual, and technical equivalence. Lastly, 15 participants completed the Spanish version of the questionnaire to confirm that the instructions, questions, and response options were clear and easily understood.

Data collection was performed by a physical therapist trained in data management and was carried out online. Prior to the data collection, subjects reported their personal and sociodemographic information. During the first session, participants who had consented completed the HIT‐6 questionnaire. In the second session, which took place 15 days after the initial session, the same participants were asked to complete the HIT‐6 questionnaire again to evaluate test‐retest reliability.

### 2.3 Measurements

The HIT‐6 is a brief instrument comprising six items that assesses the impact of headaches in clinical practice and research environments (Kosinski et al. 2003; Yang et al. 2011; Rendas‐Baum et al. 2014). As a self‐administered questionnaire, it quantifies the influence of headache‐related pain on daily activities, with each item addressing various dimensions such as social functioning, well‐being, cognitive abilities, psychological health, and vitality (Kosinski et al. 2003; Rendas‐Baum et al. 2014). Subjects provide responses on a 5‐point Likert scale, ranging from “never” to “always,” and it is mandatory to answer all items (Martin et al. 2004). The scoring system assigns values as follows: 6 for “never,” 8 for “rarely,” 10 for “sometimes,” 11 for “very often,” and 13 for “always” (Kosinski et al. 2003; Rendas‐Baum et al. 2014). The overall HIT‐6 score is derived from the sum of the six items (Martin et al. 2004; Rendas‐Baum et al. 2014), with scores ranging from 36, indicating minimal impact, to 78, reflecting a maximum impact. The scores are categorized into four distinct groups: scores of 49 or lower suggest little to no impact, scores between 50 and 55 indicate a moderate impact, scores from 56 to 59 represent a substantial impact, and scores of 60 or higher signify a severe impact (Martin et al. 2004; Rendas‐Baum et al. 2014).

To assess convergent validity with the HIT‐6 questionnaire, the Spanish version of the Migraine Disability Assessment (MIDAS) and the 12‐item Short‐Form Health Survey (SF‐12) were utilized. The MIDAS was specifically used to quantify migraine‐related disability (Rodríguez‐Almagro et al. 2020). It exhibited excellent reliability, with an intraclass correlation coefficient (ICC) of 0.81 (95% CI = 0.63–0.90; *p* < 0.001), and demonstrated good internal consistency, as evidenced by a Cronbach's alpha of 0.797 (Rodríguez‐Almagro et al. 2020).

The SF‐12 questionnaire was utilized to assess quality of life (Vilagut et al. 2008). Comprising 12 items, the SF‐12 generates two summary scores: The Physical factor summary (PCS‐12) and the Mental factor summary (MCS‐12). Both summary factors demonstrated high internal consistency, with Cronbach's alpha values of 0.85 for the PCS‐12 and 0.78 for the MCS‐12 (Vilagut et al. 2008).

### 2.4 Statistical Analysis

The analysis and management of the dataset were carried out employing IBM SPSS Statistics software, specifically version 28.0.1.0 (142) (SPSS Inc., Chicago, IL). Categorical variables were summarized using frequencies and percentages, whereas continuous variables were represented by their means and standard deviations (SD). A p‐value threshold of less than .05 was established to determine statistical significance.

In relation to construct validity, a principal component analysis (PCA) with varimax rotation was performed with the aim of identifying the underlying structure of the questionnaire variables and reducing the dimensionality. Before proceeding with the PCA, the Bartlett's sphericity test was conducted to confirm that the sample was adequate. To assess the suitability of the correlation matrix for conducting factor analysis, the Kaiser‐Meyer‐Olkin (KMO) test was performed. Results above 0.9 are considered excellent, between 0.89 and 0.80 very good, between 0.79 and 0.70 good, between 0.69 and 0.60 acceptable, between 0.59 and 0.50 bad, and below 0.50 unacceptable (Dziuban and Shirkey 1974). To determine the number of factors to retain, Kaiser's criterion (Kaiser 1960) was applied, which recommends retaining only factors with an eigenvalue greater than 1, as these factors explain a significant proportion of the variance and are considered relevant for the analysis.

The convergent validity of the HIT‐6 test was assessed in relation to the mental and physical factors of the SF‐12 questionnaire, as well as the MIDAS questionnaire, using Pearson's correlation coefficient. Correlation coefficients exceeding 0.5 were interpreted as indicating a strong correlation, while values ranging from 0.30 to 0.50 were considered to reflect a moderate correlation. Coefficients below 0.30 were classified as indicative of a weak correlation (Cohen 1988). Furthermore, following the recommendations of Cohen (Cohen 1988) and Nunnally and Bernstein (Nunnally and Bernstein 1994), correlations above 0.3 were interpreted as clinically relevant, as they reflect meaningful associations in clinical practice, even if not strong, because they capture relationships influenced by multiple factors inherent to complex conditions such as migraine.

The internal consistency of the HIT‐6 test was evaluated using Cronbach's alpha coefficient. An alpha value below 0.70 was deemed indicative of poor reliability, while values between 0.70 and 0.90 were considered to reflect good internal consistency. An alpha coefficient exceeding 0.90 was interpreted as a sign of redundancy among the items (Tavakol and Dennick 2011).

A two‐way mixed‐effects model of absolute agreement was utilized to assess test‐retest reliability through the intraclass correlation coefficient (ICC) (Shrout and Fleiss 1979). ICC values below 0.40 were interpreted as indicative of low reliability, while those ranging from 0.40 to 0.74 suggested moderate reliability. Values between 0.75 and 0.89 were considered indicative of high reliability, whereas values of 0.90 or higher were classified as demonstrating excellent reliability (Shrout and Fleiss 1979).

The standard error of measurement (SEM) quantifies the variability in measurement errors associated with a particular test (Harvill 1991). It was calculated using the formula $\mathrm{SEM}=SD_{Difference}/\sqrt{2}$, where *SD_Difference_* represents the standard deviation of the score differences between the two assessment points (time 1 and time 2). (de Vet et al. 2011; Polit 2014). Additionally, the minimum detectable change (MDC) was determined to establish a threshold for confirming that any observed alterations are authentic rather than resulting from measurement inaccuracies (de Vet et al. 2011). The MDC for individual subjects (MDCind) was calculated through the formula $\mathrm{MD}C_{ind}=1.96\times \sqrt{2}\times \mathrm{SEM}$. To further substantiate the results of interventional studies utilizing the HIT‐6 test, the MDC was also calculated for mean score comparisons between intervention and control groups (MDC_group_) (de Vet et al. 2011). This was derived from the individual MDC using the formula $\mathrm{MD}C_{group}={\mathrm{MDC}}_{ind}/\sqrt{n}$, where n represents the total number of participants in the sample (de Vet et al. 2011). Additionally, Bland—Altman plots were employed to evaluate the limits of agreement, thereby providing a graphical representation of the discrepancies between measurements (Bland and Altman 1999).

## 3 Results

The study sample consisted of 100 subjects with episodic migraine, of whom 94% were female and the remaining were male. 66% of the sample reported migraine attacks lasting more than 24 h. In addition, 30–40% of the sample reported nausea, vomiting, dizziness, photophobia, and phonophobia, associated with migraine attacks. It was also observed that more than 70% of the sample suffer from an important impact as a cause of migraine. Out of the 100 migraineurs, more than 60% mentioned the fact of having a family background of migraine. All descriptive data of the sample are shown in Table 1.

**TABLE 1:** Morphological and clinical characteristic of the sample.

		Subjects with migraine (n = 100)	
Categorical		Frequency	%
Gender	Female	94	94.00
	Male	6	6.00
Physical activity	None	27	27.00
	Low	34	34.00
	Moderate	31	31.00
	High	8	8.00
Attack duration	<4h	14	14.00
	4‐24h	20	20.00
	>24h	66	66.00
Side of pain	Left	20	20.00
	Right	35	35.00
	Both	45	45.00
Nausea	Yes	38	38.00
	No	62	62.00
Vomiting	Yes	29	29.00
	No	71	71.00
Dizziness	Yes	22	22.00
	No	78	78.00
Photophobia	Yes	32	32.00
	No	68	68.00
Phonophobia	Yes	31	31.00
	No	69	69.00
Migraine backgound	Yes	61	61.00
	No	39	39.00
Impact of migraine (HIT‐6)	No Impact	5	5.00
	Mild	14	14.00
	Important	10	10.00
	Severe	71	71.00
**Continuous**		**Mean**	**SD**
Age		36.31	10.44
Height		1.64	0.07
Weight		64.50	12.24
BMI		23.76	4.02
HIT‐6 Psychological subscale		28.88	5.32
HIT‐6 Physical subscale		33.62	3.47
SF‐12 Mental factor		41.25	12.72
SF‐12 Physical factor		49.83	8.24
Disability of migraine (MIDAS)		26.90	37.15
Attack frequency (MIDAS)		11.82	14.05
Pain intensity of migraine (MIDAS)		6.76	2.52

**Abbreviations**: %, percentage; BMI, Body mass index; HIT‐6, MIDAS; SD, standard deviation; SF‐12,

**FIGURE 2:**
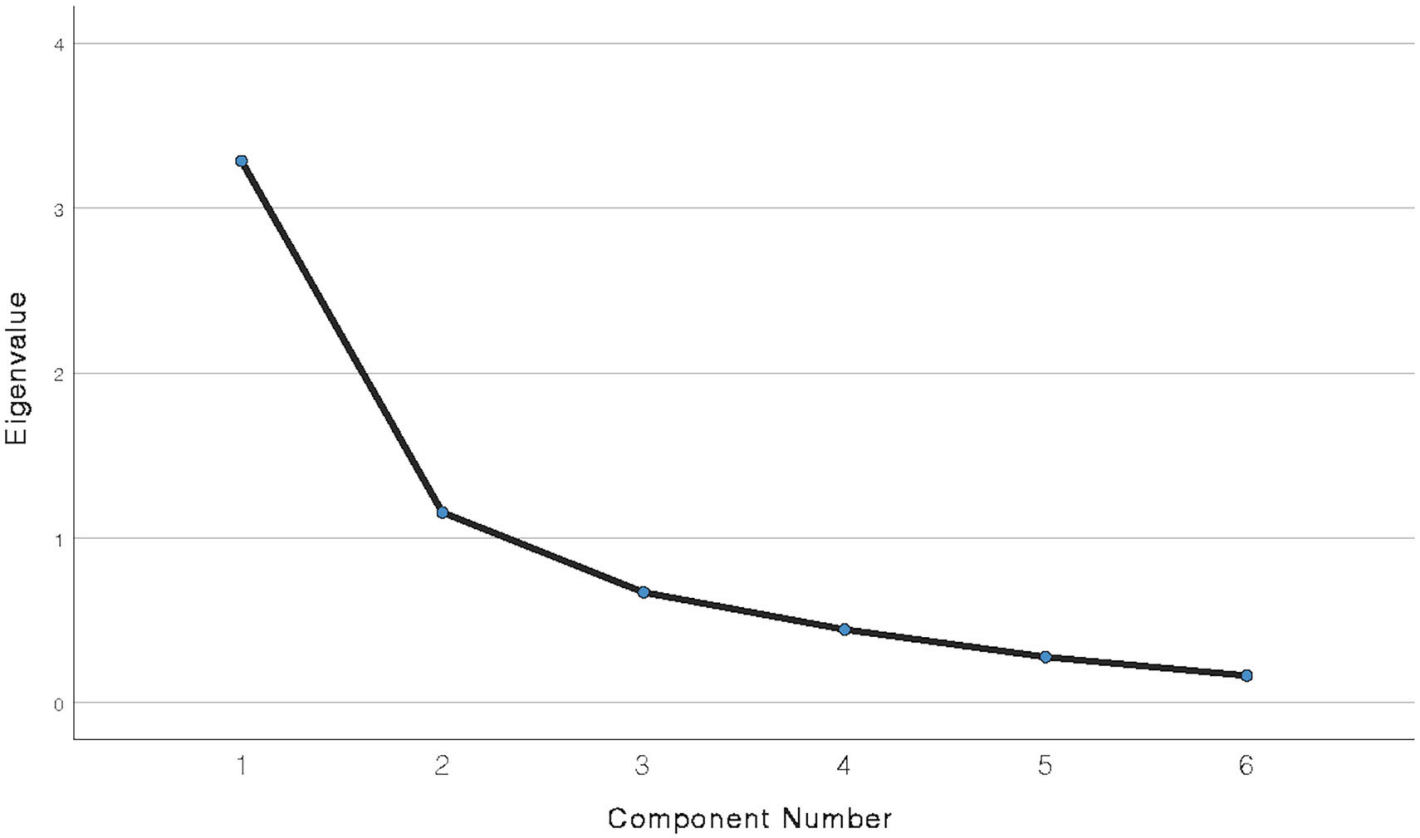
Scree plots of principal component analysis of the Spanish version of the HIT‐6 test.

The analysis carried out showed the feasibility and suitability of the PCA with statistically significant results in Bartlett´s Sphericity test (χ^2^ = 283.93; *p* < 0.001) and a KMO index of 0.713. The PCA showed a structure based on two components that explain 74.003% of the variance (Table 2 and Figure 2), with item 1 being the one that presented the lowest correlation with the whole questionnaire, with a communality of 0.518. The first component, which could reflect the impact of migraine as an indirect consequence of pain (HIT‐6 Indirect subscale), is composed of items 4, 5, and 6 (Table 3) that explain 41.32% of the variance (Table 2). The second component, which could reflect the impact of migraine as a direct consequence of pain (HIT‐6 Direct subscale), is composed of items 1, 2, and 3 (Table 3) that explain 32.68% of the variance (Table 2).

**TABLE 2:** Percentages of variance explained by the factorial analysis performed using Principal Components Analysis for the Spanish version of the HIT‐6 test.

Component	Initial Eigenvalues			Extraction sums of squared loadings			Rotation sums of squared loadings		
	Total	% of variance ^†^	Cumulative %^‡^	Total	% of variance ^†^	Cumulative %^‡^	Total	% of variance ^†^	Cumulative %^‡^
**1**	3.285	54.748	54.748	3.285	54.748	54.748	2.479	41.323	41.323
**2**	1.155	19.255	74.003	1.155	19.255	74.003	1.961	32.68	74.003
**3**	0.671	11.176	85.179						
**4**	0.445	7.42	92.6						
**5**	0.279	4.644	97.244						
**6**	0.165	2.756	100						

^†^ Percentage of variance that explains each component of the questionnaire structure.

^‡^ Total percentage of variance explained jointly by the components that compose the questionnaire structure.

**TABLE 3:** Item contribution to each component of the HIT‐6 test.

	Component	
	1	2
**Item 1**	—	0.683
**Item 2**	—	0.808
**Item 3**	—	0.834
**Item 4**	0.893	—
**Item 5**	0.880	—
**Item 6**	0.874	—

Items 1–5: Questions of the HIT‐6 test.

In the convergent validity analysis, the HIT‐6 main scale presented a strong correlation with the MIDAS questionnaire, as well as moderate correlations with physical and mental factors of the SF‐12 questionnaire. In the same line, the HIT‐6 Indirect subscale also showed a strong correlation with the MIDAS questionnaire and moderate correlations with the physical and mental factors of the SF‐12 questionnaire, while the HIT‐6 Direct subscale did not show statistically significant correlations, although it was in the limit of significance with the mental factor of the SF‐12 questionnaire (Table 4).

**TABLE 4:** Correlations among the HIT‐6 test, the MIDAS questionnaire and the physical and mental components summary of the SF‐12 questionnaire.

	HIT‐6 Main scale		HIT‐6 Indirect subscale		HIT‐6 Direct subscale	
	r	*p*‐value	r	*p*‐value	r	*p*‐value
**SF‐12 Physical**	−0.326	0.021	‐.377	0.007	0.02	0.895
**SF‐12 Mental**	−0.429	0.002	‐.435	0.002	−0.27	0.055
**MIDAS**	0.512	< 0.001	.617	< 0.001	0.09	0.539

Reliability analysis presented good internal consistency values for the HIT‐6 main scale (Cronbach´s *α* = 0.834), as well as for the HIT‐6 Indirect (Cronbach´s *α* = 0.895) and HIT‐6 Direct (Cronbach´s α = 0.725) subscales. Furthermore, the test‐retest reliability analysis was high for the HIT‐6 main Scale (ICC = 0.89; 95% CI = 0.83–0.92) and the HIT‐6 indirect subscale (ICC = 0.87; 95% CI = 0.81–0.91), while the HIT‐6 direct subscale obtained excellent test‐retest reliability (ICC = 0.90; 95% CI = 0.85–0.93) (Table 5).

**TABLE 5:** Reliability of the items and total scores of the HIT‐6 test.

Items	ICC value	Lower bound	Upper bound
**Item 1**	0.780	0.672	0.852
**Item 2**	0.857	0.787	0.904
**Item 3**	0.835	0.729	0.897
**Item 4**	0.701	0.544	0.802
**Item 5**	0.861	0.794	0.907
**Item 6**	0.829	0.746	0.885
**HIT‐6 Main Scale**	0.885	0.827	0.923
**HIT‐6 Indirect subscale**	0.871	0.814	0.912
**HIT‐6 Direct subscale**	0.899	0.849	0.933

Items 1–5: Questions of the HIT‐6 test.

**FIGURE 3:**
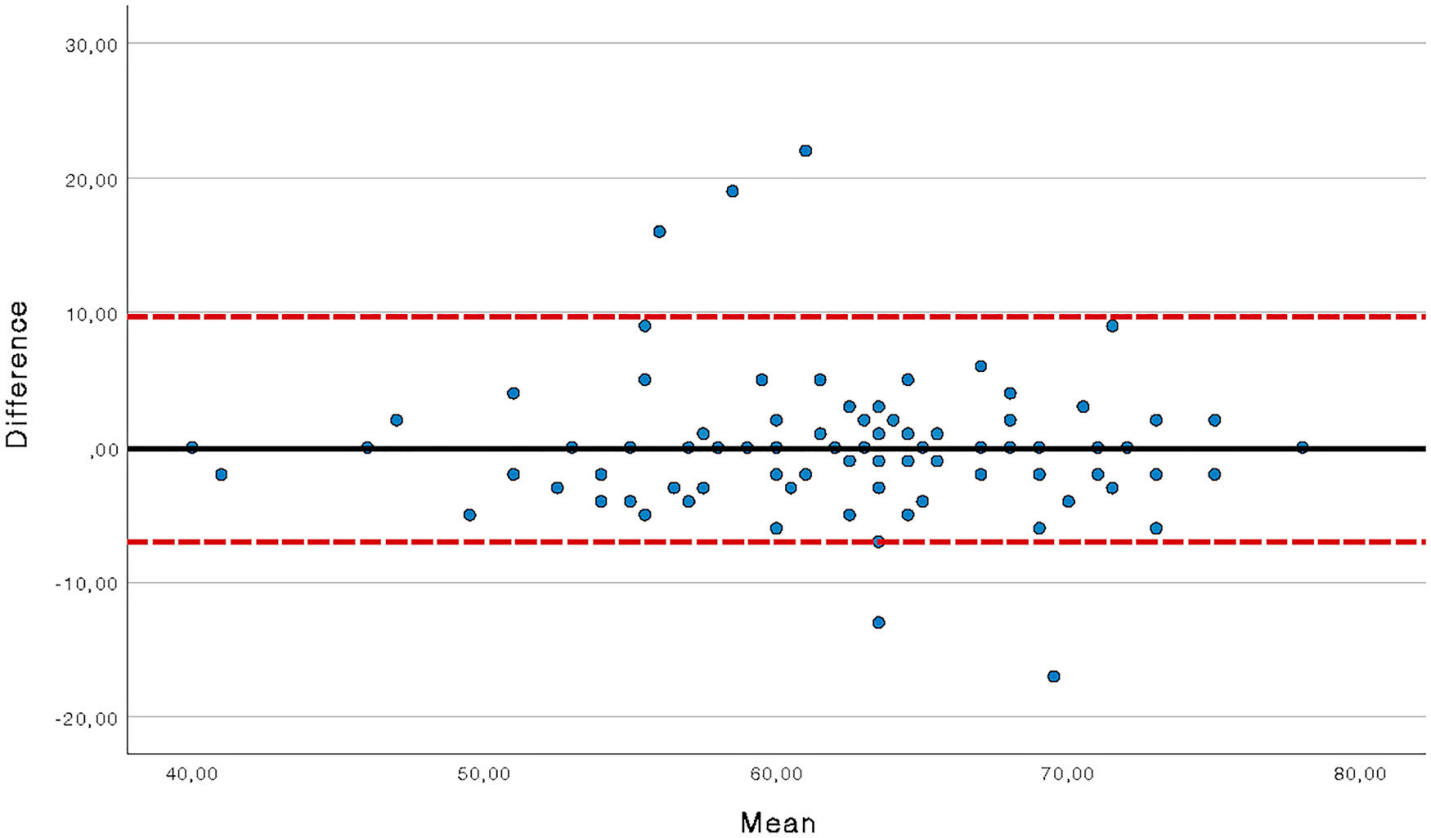
Bland—Almant plots of HIT‐6 test.

The SEM for the HIT‐6 main scale is 3.51. This means that the “real” score obtained by a patient in the HIT‐6 test can be found, with 95% confidence, between +3.51 and ‐3.51 points from the score found in the test. Moreover, the MDC_ind_ for the HIT‐6 main scale is 9.70, which means that a change in the HIT‐6 score of a subject could be considered as a true change, and not potentially a result of measurement error, if the change in HIT‐6 score is greater than 9.70 points. The MDC_group_ is 0.97 points. The limits of agreement obtained with the Bland and Altman analysis are shown in Figure 3.

## 4 Discussion

The clinical community is showing growing interest in enhancing the diagnostic and treatment approaches for migraine patients, who constitute a distinct subgroup within the general population. These individuals incur significant healthcare costs (Garrido‐Cumbrera 2019), underscoring the importance of having effective tools to measure the impact of migraine on their daily lives.

Among these tools are patient‐reported outcome measures (PROMs), which provide important information about the disease and the patient's perspective. In addition to helping us assess symptoms, health status, and quality of life of the patients (Yalinay Dikmen et al. 2023; Waliszewska‐Prosół et al. 2024).

The HIT‐6 questionnaire is a commonly used PROM and is recommended by the International Headache Society (IHS) as one of the secondary endpoints in controlled trials for both episodic and chronic migraine (Yalinay Dikmen et al. 2023; Waliszewska‐Prosół et al. 2024). It serves as a valuable tool for evaluating how migraine affects the lives of patients (Yang et al. 2011; Rendas‐Baum et al. 2014). The speed of use and ease of monitoring treatment in patients with migraine are considered advantages. However, it has two significant drawbacks: the large impact of the moment it is filled out, as the score may vary depending on whether or not the patient is experiencing a migraine episode, and the 4‐week recall period (Yalinay Dikmen et al. 2023; Waliszewska‐Prosół et al. 2024). Nonetheless, to date, there is no validated version of this questionnaire available in Spanish. This gap in resources underscores the importance of conducting a validation study.

Our findings from a population with episodic migraine revealed a two‐component structure, demonstrating good internal consistency. Additionally, we observed high to excellent test‐retest reliability and a strong correlation with the MIDAS questionnaire during the convergent validity analysis. Consequently, these results suggest that the Spanish version of the HIT‐6 questionnaire is a valid and reliable tool for measuring the impact of episodic migraine on patients' daily lives.

Unlike the original structure (Kosinski et al. 2003), our principal component analysis (PCA) revealed two distinct components that explained nearly 75% of the variance. The identification of a two‐factor structure suggests that migraine affects in a complex and multifaceted manner. The first component encompasses items associated with the indirect consequences of long‐term headaches, including fatigue, work limitations, and irritability related to migraine (items 4, 5, and 6). The second component focuses on the direct consequences of migraine, such as the frequency and intensity of pain (items 1, 2, and 3).

This two‐component structure indicates how migraine can differently influence various aspects of patients' lives, affecting their functionality and contributing to a diverse impact. It also allows for a more nuanced assessment of the effects of migraine, enabling an evaluation of both the direct consequences of pain severity and the secondary consequences due to functional impacts on patients' lives, thus offering a more thorough understanding of the disease's overall influence.

In addition to the direct consequences of migraine, various cognitive functions, including memory, attention, and speech, are negatively impacted (Vuralli et al. 2018), leading to significant disruptions in daily activities and overall quality of life (Lindbergh et al. 2016). Our findings reveal that over 70% of subjects experience a severe impact due to migraine, which aligns with the results of prior studies (Yang et al. 2011; Rendas‐Baum et al. 2014).

In our results, as in recent studies, there is a high validity and reliability of the questionnaire, particularly highlighting the internal consistency and the ability of the HIT‐6 to accurately reflect the overall impact of migraine (Haywood et al. 2021).

The Spanish adaptation of the HIT‐6 questionnaire showed solid reliability. Internal consistency for the overall scale was high, reflecting outcomes similar to those of prior studies (Yang et al. 2011; Rendas‐Baum et al. 2014), as well as more recent research (Dikmen et al. 2021). The test‐retest reliability results ranged from good to excellent, being consistent with the original method used to evaluate the migraine‐related impact. However, our study introduces a new two‐component structure to analyze the impact of pain, distinguishing between direct and indirect pain impact. This approach may offer a more detailed and specific view of the migraine impact, allowing for a better identification of experiences that affect patients more directly (such as the inability to perform activities) and those that have an emotional or indirect impact, such as irritability and fatigue.

The observations in our study are consistent with those of other recent studies (Haywood et al. 2021), but the introduction of a two‐component structure in the analysis of pain impact could provide a deeper understanding of how migraines affect both the physical and emotional aspects of patients, opening up new possibilities for clinical assessment and intervention.

The findings of this study demonstrated strong clinimetric properties for the Spanish version of the HIT‐6 questionnaire, indicating that it is a reliable and valid tool for measuring migraine impact. However, the study has some limitations. Despite the high prevalence of migraine in the general population, our results are specific to the sample analyzed and are not generalizable to other forms of migraine, such as chronic migraine, being applicable only to episodic cases. Additionally, due to cultural variations, these findings may only be relevant to the Spanish population, as cultural differences can influence how people interpret and respond to the questionnaire. Furthermore, potential biases could arise, such as the selection of participants based on accessibility, which may introduce selection bias, affecting the sample's diversity and limiting the representativeness and generalizability of the results. Another potential limitation of this study is that different subpopulations of patients, particularly those with varying migraine frequencies, may interpret and respond to the questionnaire differently. Specifically, patients experiencing more frequent episodes may perceive certain items differently compared to those with sporadic migraines, which could affect the reliability and validity of the responses, as well as the generalizability of the results.

## 5 Conclusions

Our study indicated that the Spanish version of the HIT‐6 questionnaire (see Supplementary Material in Supporting Information section) is a valid and reliable tool to measure the impact on the quality of life of patients with episodic migraine. Supporting Information

## Author Contributions

**Yaiza Colorado‐Martín**: conceptualization, investigation, and writing–original draft. **Daniel Pecos‐Martín**: conceptualization, writing–review and editing, visualization, and validation**. Nerea de Miguel‐Hernando**: investigation, writing–original draft, visualization, and validation**. Rubén Cámara‐Calmaestra**: investigation, methodology, and supervision**. Daniel Rodríguez‐Almagro**: formal analysis, writing–original draft, and writing–review and editing**. Alejandro Ferragut‐Garcías**: investigation, visualization, and validation**. Eduardo Castro‐Martín**: investigation, methodology, and supervision**. Alexander Achalandabaso‐Ochoa**: conceptualization, writing–original draft, writing–review and editing, supervision, and project administration. All authors have read and agreed to the published version of the manuscript.

## Conflicts of Interest

The authors declare no conflicts of interest.

### Peer Review

The peer review history for this article is available at https://publons.com/publon/10.1002/brb3.70515

## Supporting information



Supporting Information

## Data Availability

The data that support the findings of this study are available from the corresponding author upon reasonable request.
