# Supplementary material for: Validation of the Spanish Version of the Headache Impact Test (HIT‐6) in Patients With Episodic Migraine
Source: Brain Behav. 2025 May 5;15(5):e70515. doi: 10.1002/brb3.70515 (PMC12050642; doi:10.1002/brb3.70515)

**Escala HIT-6**  FECHA: / / .

Este cuestionario fue diseñado para ayudarlo a describir y comunicar cómo se siente y lo que no puede hacer debido a los dolores de cabeza.

INSTRUCCIONES: Para completar el cuestionario, marque una casilla para cada pregunta.

1. Cuando tiene dolor de cabeza, ¿con qué frecuencia es intenso?


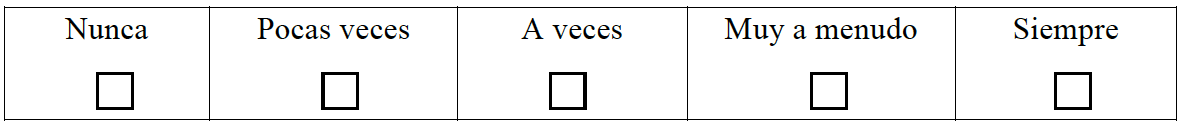


2. ¿Con qué frecuencia los dolores de cabeza le dificultan realizar actividades de la vida diaria como son las tareas del hogar, el trabajo, los estudios o las actividades sociales?


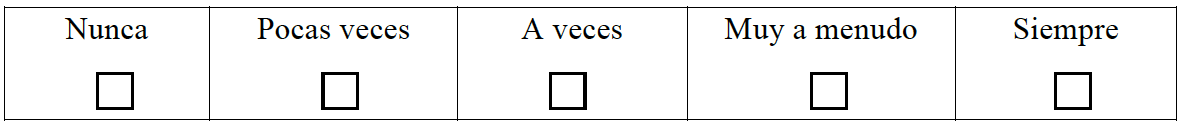


3. Cuando tiene dolor de cabeza, ¿con qué frecuencia querría tumbarse o acostarse en la cama?


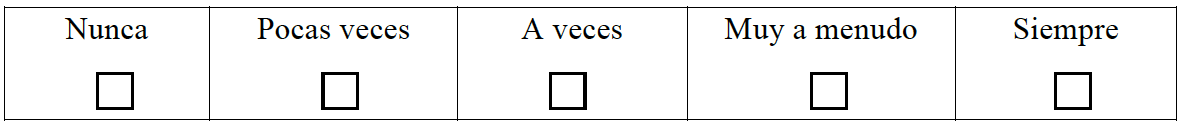


4. En las últimas 4 semanas, ¿con qué frecuencia ha sentido que el dolor de cabeza le impide trabajar o llevar a cabo sus actividades de la vida diaria debido al cansancio?
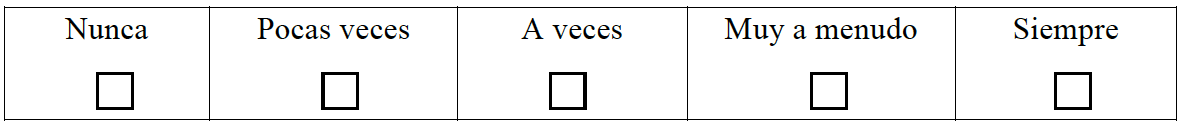


5. En las últimas 4 semanas, ¿con qué frecuencia se ha sentido molesto/a o irritado/a debido a los dolores de cabeza?
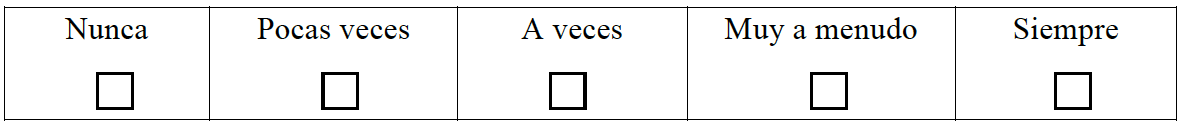


6. En las últimas 4 semanas, ¿con qué frecuencia los dolores de cabeza han limitado la capacidad de concentración en el trabajo o las actividades de la vida diaria?


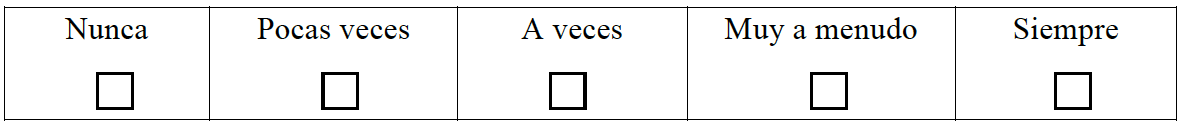

Supplement: Supplementary file 1 — Supporting Information [file BRB3-15-e70515-s001.docx]
